# First case report of thyroid abscess caused by *Helicobacter cinaedi* presenting with thyroid storm

**DOI:** 10.1186/s12879-019-3808-7

**Published:** 2019-02-15

**Authors:** Tomohiro Takehara, Tetsuo Tani, Ken Yajima, Mako Watanabe, Yoshihito Otsuka, Hidefumi Koh

**Affiliations:** 1 Division of Pulmonary Medicine, Department of Medicine, Keio University School of Medicine, Tokyo, Japan; 2 Division of Pulmonary Medicine, Department of Internal Medicine, Federation of National Public Service Personnel Mutual Aid Associations, Tachikawa Hospital, 4-2-22 Nishikicho, Tachikawa, Tokyo, 190-8531 Japan; 3 Division of Diabetes, Endocrinology and Metabolism, Department of Internal Medicine, Federation of National Public Service Personnel Mutual Aid Associations, Tachikawa Hospital, Tokyo, Japan; 4 Department of Microbiological Laboratory, Federation of National Public Service Personnel Mutual Aid Associations, Tachikawa Hospital, Tokyo, Japan; 5 Department of Clinical Laboratory, Kameda Medical Center, Chiba, Japan

**Keywords:** *Helicobacter cinaedi*, Thyroid abscess, Basedow’s disease, Thyroid storm

## Abstract

**Background:**

*Helicobacter cinaedi* is a microaerobic Gram-negative spiral-shaped bacterium that causes enteritis, cellulitis, and bacteremia in both immunocompromised and immunocompetent patients. While there have been increasing numbers of reported *H. cinaedi* infections recently, there has been no thyroid abscess case caused by *H. cinaedi* presenting with thyroid storm.

**Case presentation:**

A 50-year-old Japanese man presented with a 9-day history of high fever associated with palpitations, dry cough, and chronic diarrhea. The patient had a history of Basedow’s disease that had been treated with thiamazole in the past. During the current episode, the patient was diagnosed with thyroid storm and treated accordingly. The blood culture taken on admission was positive for *H. cinaedi*. This finding was confirmed by matrix-assisted laser desorption ionization-time of flight mass spectrometry (MALDI-TOFMS). A systemic computed tomography (CT) scan revealed a thyroid abscess as the site of infection and cause of the bacteremia. The 16S rRNA gene sequencing identified the pathogen of thyroid abscess as *H. cinaedi*. Clinical symptoms and laboratory data normalized on admission day 7 after treatment with both effective antibiotics and antithyroid drugs.

**Conclusions:**

The case study described a patient with a history of Basedow’s disease that presented with a thyroid abscess caused by *H. cinaedi* with symptoms similar to those of thyroid storm. While this bacterium has been implicated in other infections, we believe this is the first time the bacteria has been documented to have caused a thyroid abscess.

**Electronic supplementary material:**

The online version of this article (10.1186/s12879-019-3808-7) contains supplementary material, which is available to authorized users.

## Background

*Helicobacter cinaedi* is a microaerobic Gram-negative spiral-shaped bacterium first documented as a cause of gastrointestinal symptoms in patients with human immunodeficiency virus (HIV) in 1984 [1]. Soon after its discovery, *H. cinaedi* was mostly found in HIV patients presenting with gastrointestinal disease, but was subsequently detected in other immunosuppressive conditions such as hematological disease, cancer, chronic liver disease, asplenia, and patients being treated with immunosuppressive drugs [2]. In several recent reports, *H. cinaedi* infection has also been identified in immunocompetent people [3, 4]. *H. cinaedi* is known to be the causative organism of bacteremia [5], cellulitis [6], enteritis [7], arthritis [8], meningitis [9], infective endocarditis [10], vertebral osteomyelitis [11], and kidney cyst infection [12]. *H. cinaedi* is a slow-growing bacterium in culture and difficult to diagnose [13]. Additionally, the standard antibiotic therapy for *H. cinaedi* infection has not yet been determined. Patients may suffer reoccurring *H. cinaedi* infection if the treatment time is not long enough [14].

It is extremely rare for a thyroid abscess to be caused solely by a bacterial infection because the thyroid gland anatomically and physiologically forms a protective barrier to infection [15]. Previous reports state that thyroid abscesses are usually caused by anatomical abnormalities like pyriform sinus fistula [16] or due to ingestion a foreign body that subsequently damages the thyroid, such as a migratory fish bone [17]. The causative pathogens in these cases are mainly Gram-positive organisms such as *Staphylococcus* and *Streptococcus* species [18].

This case study presents the first known case of a thyroid abscess caused by *H. cinaedi* in an immunocompetent patient with symptoms similar to thyroid storm.

## Case presentation

A 50-year-old Japanese man presented to our hospital with a 9-day history of a high fever associated with palpitations and dry cough. He denied a sore throat, cervical pain, dyspnea, and stomachache. He had a 7-year history of Basedow’s disease previously treated with thiamazole, but had discontinued the medication more than one year before the current admission. The patient had chronic mild diarrhea and had lost 20 kg in the preceding 6 months. Additional past medical history included childhood asthma. The only regular medication taken by the patient was the discontinued thiamazole. The patient had smoked 1 pack of cigarettes a day for 30 years, but drank no alcohol. The only sexual contact was with his wife. He denied any sick contacts or recent travel.

**Fig. 1:**
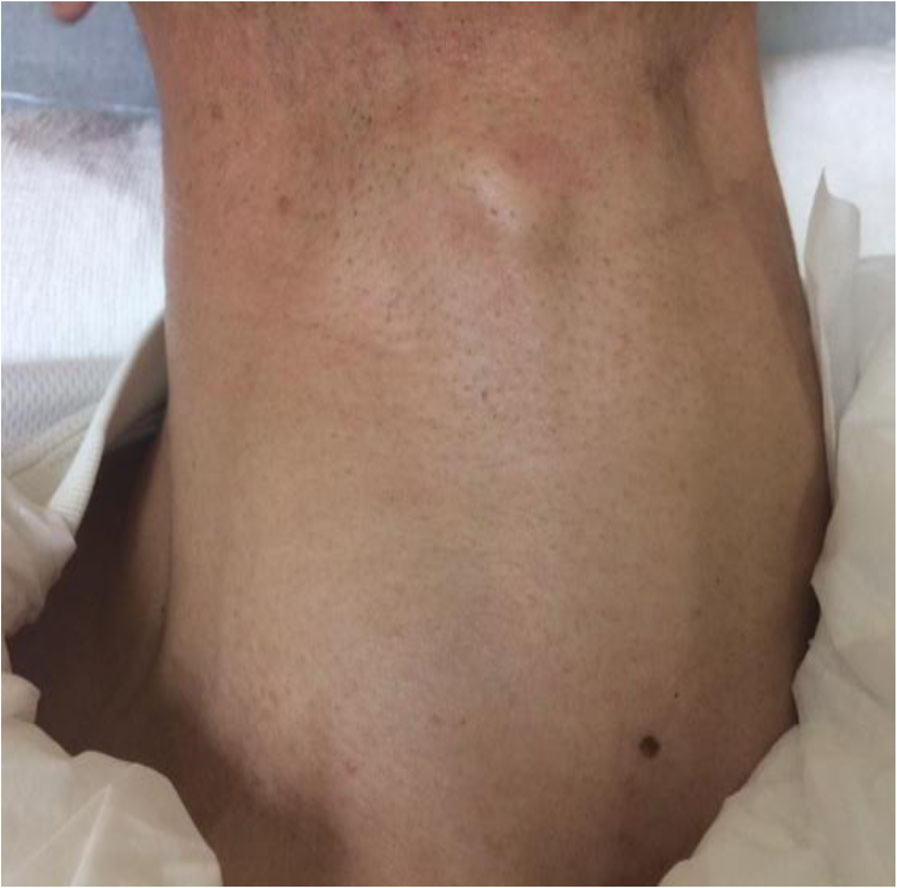
The thyroid gland on admission day. It was diffusely enlarged but had a normal cutaneous appearance

On physical examination, the patient appeared anxious and was febrile (temperature, 39.1 °C). His blood pressure was 129/77 mmHg, pulse 131 beats per minute, and respiratory rate 34 breaths per minute with an oxygen saturation of 97% on room air. He was alert and had an exophthalmos. On palpation, the thyroid gland was soft and diffusely enlarged, but non-tender with no skin redness or warmth (Fig. 1). His cardiovascular examination revealed tachycardia and the lungs were clear to auscultation. His bowel sounds were hyperactive, but the abdomen was non-tender. Neurological examination yielded completely normal findings and no skin rash was present.

Initial laboratory results showed leukocytosis (11,360 white blood cells (WBC) /μL with neutrophils at 78.6%) and hyperthyroidism with TSH < 0.021 μIU/mL, F-T3 > 30.00 pg/mL, F-T4 at 8.88 ng/mL, and TSH receptor antibody 42.2 IU/L. Other significant laboratory findings were C-reactive protein (CRP) at 5.07 mg/dl, and glycated hemoglobin (HbA1c) at 5.6%. Test results for HBs antigen, HBs antibody, HCV antibody, and HIV antibody were not remarkable. There were normal findings on the urinalysis and chest X-ray findings. Echocardiography showed normal ejection fraction (64%) and no signs of valve vegetation.

**Fig. 2:**
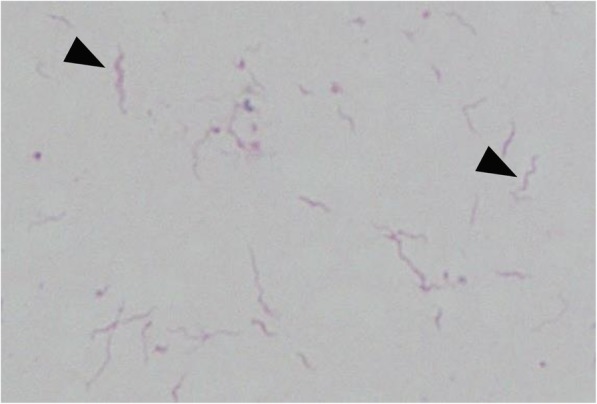
The Gram staining (original magnification × 1000) of blood culture. There were Gram-negative spiral and curved bacteria (black arrowhead)

After admission, the patient was initially diagnosed with thyroid storm (due to tachycardia, diarrhea, and thyroid hormone levels) and treatment with thiamazole (30 mg/day), hydrocortisone (200 mg/day), and potassium iodide (100 mg/day) was initiated. Due to the high fever and dry cough, he was also treated with ceftriaxone (CTRX) (2 g/day). Sputum and blood cultures were taken to determine the causative agent. After 80 h post admission, Gram-negative bacteria were cultured in two sets of aerobic blood culture bottles. Blood cultures were processed through the BACTEC FX system (Becton Dickinson, Sparks, Maryland). Gram staining of the cultured bacteria showed a spiral and curved shape, which was morphologically compatible with *H. cinaedi* (Fig. 2). This identification was confirmed via matrix-assisted laser desorption ionization-time of flight mass spectrometry (MALDI-TOFMS). MALDI-TOFMS analysis was performed, using a MALDI Biotyper system (Bruker Daltonics, Bremen, Germany) as previously described [19]. The pathogen was identified as *H. cinaedi* with a score of 2.0. The score of ≥2.0 was useful for identification to the species level. The sputum culture yielded no positive findings, including for acid-fast bacteria.

**Fig. 3:**
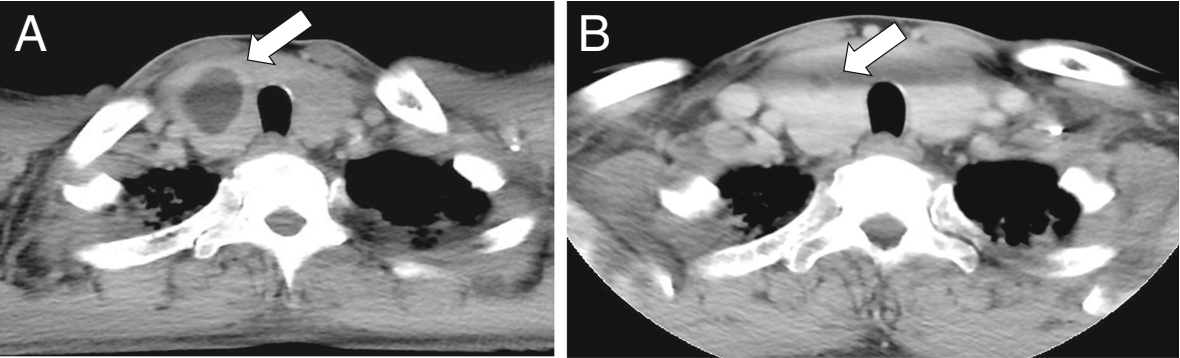
The thyroid gland findings on computed tomography (CT) scan. **a**. The systemic contact-enhanced CT scan on admission day 4 showing enlarged thyroid gland and low-density area in the right lobe (white arrow). **b**. The systemic contact-enhanced CT scan of the thyroid gland 5 months after admission showing the small remains of the thyroid abscess in the right lobe (white arrow)

**Fig. 4:**
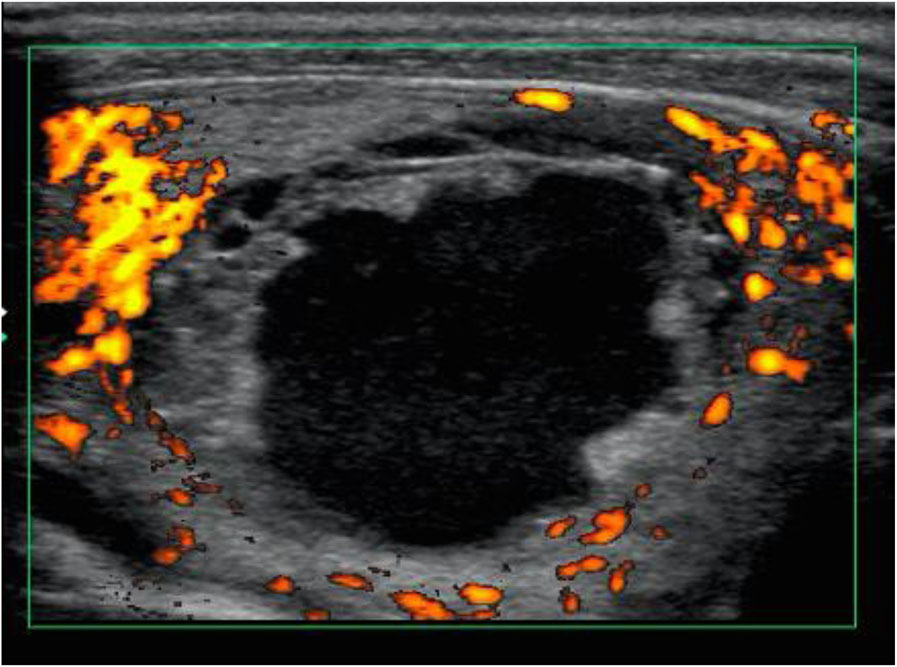
Ultrasound examination of the thyroid gland on admission day 4 showing a thyroid nodule in the right lobe surrounded by enriched thyroid vessels

**Fig. 5:**
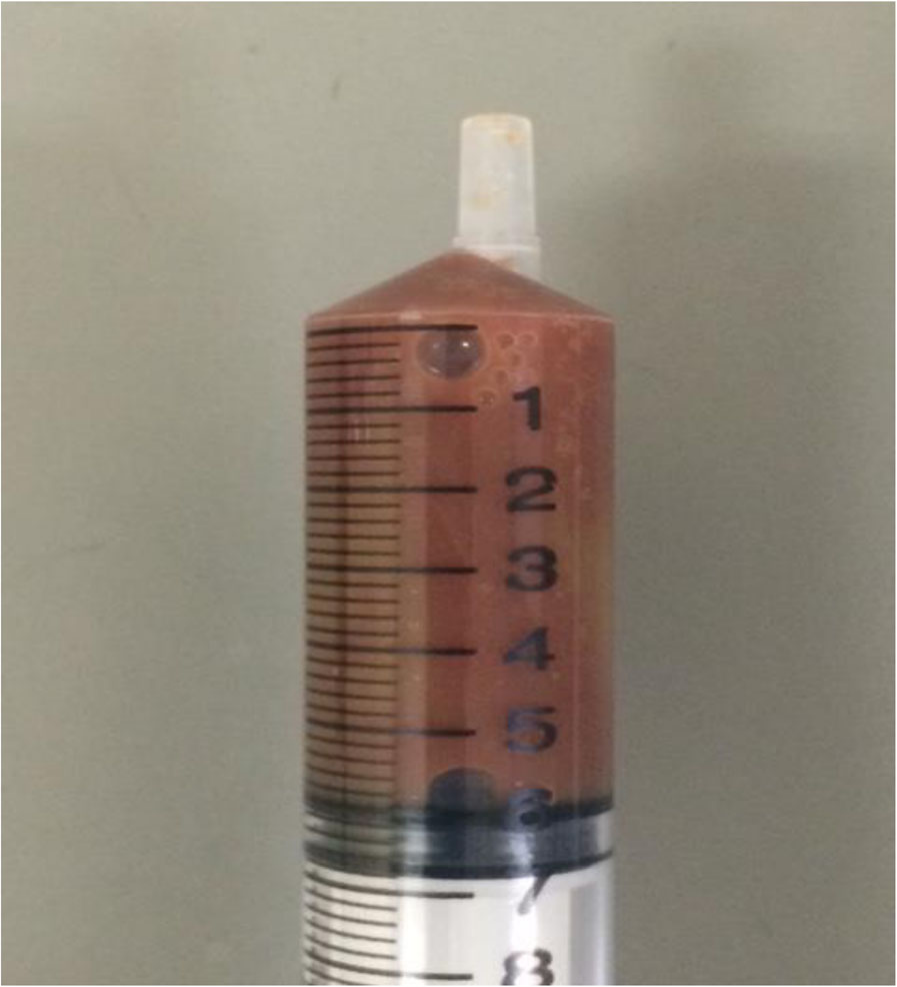
The reddish purulent liquid drained from the thyroid nodule in the right lobe

To determine the origin of the infection, we performed a whole-body contrast-enhanced CT scan and found a thyroid nodule in the right lobe (Fig. 3a). There was no other infection site identified and the size of the spleen was normal. The thyroid gland ultrasound examination showed high speed velocity in the thyroid vessels and a right lobe thyroid nodule that measured approximately 36 × 38 × 28 mm (Fig. 4). When the patient was first diagnosed with Basedow’s disease 7 years previously, there was no thyroid cyst present at that time. Fine-needle aspiration biopsy revealed that the thyroid nodule contained a reddish purulent liquid with a total volume of 6 ml (Fig. 5). Laboratory examination of the aspirated liquid revealed a total cell count of 40,000/μL with polymorphonuclear leukocytes 14,667/μL, lactate dehydrogenase 16,990 IU/L, protein 3.5 g/dL, pH level of 7.039, and glucose at 31 mg/dl. These findings were compatible with a diagnosis of bacterial abscess. Likely due to CTRX administration upon admission, the Gram staining and culture of the specimens yielded no bacteria including acid-fast bacteria. Hence, we analyzed the liquid via broad-range 16S rRNA gene polymerase chain reaction (PCR). Nucleic acids were extracted from the liquid using a QIAamp DNA Mini Kit (Qiagen, Limburg, Netherlands) as specified by the manufacturer, and fragments of the 16S rRNA gene were PCR-amplified using universal primers. The PCR products were sequenced using an Applied Biosystems 3130xl Genetic Analyzer (Thermo Fisher Scientific, Waltham, MA, USA) with the primers used for PCR amplification. The amplified 16S rRNA sequence had the highest similarity to that of *H. cinaedi* [1441/1451 (99.31%) base pair matches in BLAST, and 1436/1438 (99.51%) base pair matches in Ez Taxon]. An additional file showed the result of the sequence in more detail [see Additional file 1]. The patient was then diagnosed with *H. cinaedi* bacteremia and thyroid abscess complicated by uncontrolled Basedow’s disease.

After the determination of the causative pathogen, we changed antibiotics from CTRX to amoxicillin (AMPC) at 1.5 g/day. On admission day 4, the patient’s high fever, tachycardia, and diarrhea were improved. The serum levels of CRP and thyroid hormone were also improving. Blood cultures were drawn again on admission day 10 and were then negative. A laryngeal endoscopy was performed to check for any anatomical abnormalities and the findings were normal. The patient was discharged on admission day 24, and continued taking thiamazole (30 mg/day) and AMPC (1.5 g/day).

On follow-up in the outpatient clinic, the patient received both a thyroid echography and contrast-enhanced CT scan several times to ensure the abscess had not relapsed (Fig. 3b). The AMPC was taken for 6 months in total. There were no subsequent recurrences of thyrotoxic symptoms or infection. After the inflammation and infection were cleared, esophagography detected no anatomic anomalies such as pyriform sinus fistula. Seven months after diagnosis, the patient underwent a total thyroidectomy for the treatment of Basedow’s disease and to prevent recurrence of a thyroid abscess. The histopathology of the removed thyroid showed it was benign and did not have signs of the thyroid abscess. The patient is now taking levothyroxine for the management of hypothyroidism.

## Discussion and conclusions

To the best of our knowledge, this is the first documented case of thyroid abscess caused by *H. cinaedi* presenting with symptoms similar to thyroid storm. The patient’s clinical symptoms and thyroid hormone values were suggestive of thyroid storm, but could also signify a systemic inflammatory response syndrome induced by bacteremia. Steroid administration for treatment of thyroid storm masks fever and suppresses immunity, which can worsen infectious disease processes. Consequently health care providers should be cognizant of the possibility of bacterial infections in febrile patients with Basedow’s disease and the need for blood cultures to definitively diagnose the condition. In this case study, the patient was considered immunocompetent. He would not have been expected to be at risk for *H. cinaedi* infection. Araoka et al. reported that the rate of positive stool cultures was about 50% in cases of *H. cinaedi* bacteremia, suggesting the possibility of bacterial translocation [20]. We initially thought the patient’s diarrhea was due to thyroid storm, so failed to obtain a stool culture. We speculate that the *H. cinaedi* bacteremia was caused by chronic diarrhea due to uncontrolled Basedow’s disease, which triggered intestinal bacterial translocation. *H. cinaedi* bacteremia subsequently led to the thyroid abscess.

Acute suppurative thyroiditis (AST) or thyroid abscess is a rare infectious disease, representing less than 1% of all thyroid diseases because the thyroid gland has rich vascularity, a protective capsule, and high iodine content [21]. AST and thyroid abscess usually present with an acutely enlarging neck mass. The main causes are pyriform sinus fistula related to anomalies of the third and fourth branchial pouches [22], following neoplastic thyroid nodule [21, 22], Hashimoto thyroiditis [23], and penetration of the thyroid gland by foreign bodies such as fish bones [17]. Underlying comorbidities including tuberculosis, diabetes mellitus, and HIV infection appear to increase the risk of developing thyroid abscess. *Staphylococcus* and *Streptococcus* species are the major causative organisms of AST [18], and *Salmonella* [24], *Mycobacterium tuberculosis* [25] have also been reported in rare cases.

Additionally, this is also the first case of thyroid abscess caused by *H. cinaedi* in a Basedow’s disease patient with normal neck anatomy. At the time of diagnosis of Basedow’s disease, thyroid gland echography revealed no thyroid cyst or nodule. The clinical manifestations of this case are extremely rare as the patient had no pain or outward sign of infection at the thyroid or in surrounding tissue. This implies that the thyroid abscess was caused by *H. cinaedi* bacteremia, not by bacterial entry from the outer skin. The broad-range PCR method is robust especially for detecting and identifying the causative pathogens of culture-negative infections such as this case [26].

As noted, the patient was not adhering to the recommended medication regime to control the Basedow’s disease at the time of admission. Additionally, since thyroid abscess can be a risk factor for the recurrence of thyroid storm, we considered him an appropriate candidate for thyroidectomy. Prior to surgery, the thyroid hormone levels had to be controlled in order to downsize the thyroid gland to minimize intraoperative blood loss. For this reason, the duration of antibiotic administration was much longer than that of a previously reported case [27]. There was no evidence of thyroid abscess at the surgical site, so the duration of antibiotic administration appeared to be sufficient. We showed that *H. cinaedi*-induced thyroid abscess could be cured by AMPC combined with drainage of the abscess and control of thyroid hormone levels. There are currently no recommended guidelines on the choice of antibiotic therapy and drug susceptibility testing for *H. cinaedi*. The agar dilution method is generally used for these bacterial strains; however, we did not have the results and treated the case by AMPC according to past reports [6, 28].

In conclusion, this case is quite meaningful in its clinical manifestation, causative pathogen, infection site, and treatment. Further reports are necessary to clarify the association between thyroid abscess and *H. cinaedi* bacteremia.

## Additional file

Additional file 1: The 16S rRNA sequence of the isolated bacteria (DOCX 12 kb)

## Abbreviations

AMPC: Amoxicillin
AST: Acute suppurative thyroiditis
CRP: C-reactive protein
CT: Computed tomography
CTRX: Ceftriaxone
F-T3: Free triiodothyronine
F-T4: Free thyroxine
*H. cinaedi*: *Helicobacter cinaedi*
HbA1c: glycated hemoglobin
HBs: Hepatitis B virus
HCV: Hepatitis C virus
HIV: Human immunodeficiency virus
PCR: Polymerase chain reaction
TSH: Thyroid stimulating hormone
