# Supplementary material for: First case report of thyroid abscess caused by Helicobacter cinaedi presenting with thyroid storm
Source: BMC Infect Dis. 2019 Feb 15;19:166. doi: 10.1186/s12879-019-3808-7 (PMC6377776; doi:10.1186/s12879-019-3808-7)
Supplement: Supplementary file 1 — The 16S rRNA sequence of the isolated bacteria (DOCX 12 kb) [file 12879_2019_3808_MOESM1_ESM.docx]

**Additional file 1**

CATGGCTCAGAGTGAACGCTGGCGGCGTGCCTAATACATGCAAGTCG AACGATGAAGCTTTTAGCTTGCTAGAAGTGGATTAGTGGCGCACGG GTGAGTAATGCATAGGTTATGTGCCCTTTAGTCTGGGATAGCCACTGG AAACGGTGATTAATACTGGATACTCCCTACGGGGGAAAGGTTTTTCG CTAAAGGATCAGCCTATGTCCTATCAGCTTGTTGGTGAGGTAATGGCT CACCAAGGCTATGACGGGTATCCGGCCTGAGAGGGTGATCGGACAC ACTGGAACTGAGACACGGTCCAGACTCCTACGGGAGGCAGCAGTAG GGAATATTGCTCAATGGGGGAAACCCTGAAGCAGCAACGCCGCGTG GAGGATGAAGGTTTTAGGATTGTAAACTCCTTTTGTAAGAGAAGATT ATGACGGTATCTTACGAATAAGCACCGGCTAACTCCGTGCCAGCAGC CGCGGTAATACGGAGGGTGTAAGCGTTACTCGGAATCACTGGGCGT AAAGAGTGCGTAGGCGGGTAGTCAAGTCAGATGTGAAATCCTGTAG CTTAACTACAGAACTGCATTTGAAACTGACTATCTAGAGTATGGGAGA GGTAGGTGGAATTCTTGGTGTAGGGGTAAAATCCGTAGAGATCAAG AGGAATACTCATTGCGAAGGCGACCTGCTGGAACATTACTGACGCTG ATGCGCGAAAGCGTGGGGAGCAAACAGGATTAGATACCCTGGTAGT CCACGCCCTAAACGATGAATGCTAGTTGTTGCCCTGCTTGTCAGGGC AGTAATGCAGCTAACGCATTAAGCATTCCGCCTGGGGAGTACGGTCG CAAGATTAAAACTCAAAGGAATAGACGGGGACCCGCACAAGCGGTG GAGCATGTGGTTTAATTCGAAGCTACGCGAAGAACCTTACCTAGGCT TGACATTGATAGAATCTGCTAGAGATAGCGGAGTGCTGGCTTGCCAG AGCTTGAAAACAGGTGCTGCACGGCTGTCGTCAGCTCGTGTCGTGA GATGTTGGGTTAAGTCCCGCAACGAGCGCAACCCTCGTCCTTAGTTG TTAGCAGTTCGGCTGAGCACTCTAAGGAGACTGCCTTCGCAAGGAG GAGGAAGGTGAGGACGACGTCAAGTCATCATGGCCCTTACGCCTAG GGCTACACACGTGCTACAATGGGACATACAAAAAGATGCAATATCGC GAGATGGAGCAAATCTCTAAAATGTCTCTCAGTTCGGATTGTAGTCTG CAACTCGACTACATAAAGCTGGAATCGCTAGTAATCGCAAATCAGCAA TGTTGCGGTGAATACGTTCCCGGGTCTTGTACTCACCGCCCGTCACAC CATGGGAGTTGTATTCGCCTTAAGTCGGGATACTAAATTGGTTACCGC CCACGGCGGATGCAGCGACTGGGGTGAAGTCGTAACAAGGTAACCGTAAT
